# Use and perceived benefits and barriers of QSAR models for REACH: findings from a questionnaire to stakeholders

**DOI:** 10.1186/1752-153X-6-159

**Published:** 2012-12-18

**Authors:** Claire Mays, Emilio Benfenati, Simon Pardoe

**Affiliations:** 1 Symlog, 262 rue St Jacques, Paris 75005, France; 2 Istituto di Ricerche Farmacologiche “Mario Negri”, Via La Masa 19, Milano 20156, Italy; 3 PublicSpace Ltd, Bletherbeck House, Ulverston LA12 8DB, UK

## Abstract

The ORCHESTRA online questionnaire on “benefits and barriers to the use of QSAR methods” addressed the academic, consultant, regulatory and industry communities potentially interested by QSAR methods in the context of REACH. Replies from more than 60 stakeholders produced some insights on the actual application of QSAR methods, and how to improve their use.

Respondents state in majority that they have used QSAR methods. All have some future plans to test or use QSAR methods in accordance with their stakeholder role.

The stakeholder respondents cited a total of 28 models, methods or software that they have actually applied. The three most frequently cited suites, used moreover by all the stakeholder categories, are the OECD Toolbox, EPISuite and CAESAR; all are free tools.

Results suggest that stereotyped assumptions about the barriers to application of QSAR may be incorrect. Economic costs (including potential delays) are not found to be a major barrier. And only one respondent “prefers” traditional, well-known and accepted toxicological assessment methods.

Information and guidance may be the keys to reinforcing use of QSAR models. Regulators appear most interested in obtaining clear explanation of the basis of the models, to provide a solid basis for decisions. Scientists appear most interested in the exploration of the scientific capabilities of the QSAR approach. Industry shows interest in obtaining reassurance that appropriate uses of QSAR will be accepted by regulators.

## Finding

The paper shows results on a questionnaire on the use of in silico methods for the prediction of the properties of chemical substances. All respondents are interested on the in silico methods and there is no a priori barrier. However, responses reveal different priorities on the use and preferences on the properties modelled, depending on the stakeholders: regulators, scientists, or industry representatives. Freely available models, such as EPISuite, OECD Toolbox, and CAESAR, are more frequently used.

### Introduction

In recent years the EU has funded research into developing computer-based methods for evaluating the toxicity of chemicals, called QSAR or ‘Quantitative structure-activity relationship’ models. These computerized models are potentially important in making it possible to evaluate large numbers of chemicals (as required by the EU REACH legislation) while also reducing the numbers of tests on animals.

The ORCHESTRA project [1] is funded by the EC to communicate some of the research findings to regulators, industry users and others. The intention is to promote the wider understanding, awareness and appropriate use of QSAR methods.

Within ORCHESTRA we conducted a survey of stakeholders to identify the perceived benefits, barriers and needs in relation to the regulatory use of QSAR methods. We produced a questionnaire addressing potential and actual users of QSAR models: regulatory bodies, industry, scientists and consultants. Responses to the questionnaire provide insight into:

– The current awareness of, and interest in, QSAR methods

– The perceived benefits and attractions of QSAR methods

– The current barriers to the use of QSAR methods

– The current professional and policy needs

– Stakeholders’ current sources of information on *in silico* methods in toxicology.

Note that for simple convenience, the terms “QSAR” and “*in silico*”, or “models” and “methods”, are used indiscriminately in this report.

A fully detailed report [2] of the questionnaire study, including aspects not considered in this brief article, may be found online at the (identical) project websites: http://www.orchestra-qsar.eu or http://www.in-silico-methods.eu.

#### Questionnaire and sample

The invitation to respond to the online questionnaire took the form of a letter in English that was circula-ted by email first to 280 potential respondents across Europe and beyond, and then to a much larger set of stakeholders. However, since the flow of responses was small, a second questionnaire was created as a quick alternative for professionals who might feel too busy to respond to a survey. Questionnaire I contained 8 questions, while Questionnaire II had only 2–4. The period of data collection was Sept 2010 – April 2011.

Both questionnaires can be viewed online at the project websites mentioned above. Questionnaire I and II yielded 33 and 29 responses respectively, 62 in total. There was no overlap between the populations replying to the two questionnaires.

The total sample is unfortunately too small to represent in detail the full population that was approached for this survey. However, some trends or groupings in response seem to emerge, and great care has been taken to interpret these at an appropriate level.

Questionnaire I asked participants to designate the context in which their research, development and/or application of *in silico* methods take place. On the basis of a careful examination of these descriptive data, three categories were constructed:

• **ACACON** – Activity takes place in an **aca**demic or **con**sultancy context. Members of this category display many different roles and stakes within the REACH process, but they are distinguished by the facts that they are not direct employees of an industrial manufacturer of chemical products, nor are they tasked as protectors of public health. ^a^*13 subjects*

• **REGUL** - Activity takes place in a governmental **regul**atory context. Members’ primary stake and national (or international) mission lie in protecting public health or the environment. *12 subjects*

• **INDUS –** Activity takes place within a commercial **indus**trial context. Members are directly employed by manufacturers of chemical products, and their company has a primary economic stake in the outcome of specific REACH dossiers. (A chemical manufacturers’ federation is also represented in this category). *8 subjects*

As stated above, no claim is made to describe stakeholder populations at large; the suggestive trends we observe in Questionnaire I replies would demand testing on a larger survey population. This said, systematically collected survey data from 33 specialists may be more solid than anecdotal impressions. Finally, the interpretations offered here are highly coherent not only with the actual professional experience of ORCHESTRA project members but moreover with the in-depth interview findings documented in other parts of the project. These findings are provided on the ORCHESTRA websites. Notably, on April 6^th^ 2011 a workshop was held entitled “REACH and QSAR: What can we learn from case studies”; the summary available online recounts needs for better understanding of the uses of QSAR models, for better inter stakeholder communication, and for training, all of which are borne out by the survey reported in the present article. Also online, the extensive video interviews of academics, consultants, regulators and industry representatives help to understand particular priorities among these different populations [http://www.orchestra-qsar.eu or http://www.in-silico-methods.eu].

#### Data presentation

Because the sample is small, after discussion with scientists and with laypersons we decided we should not refer to percentages of response in our discussion of results. Instead, an illustrative “green light” system of presentation is chosen. This system is used throughout and indicates in an intuitive way the relative strength or proportion of response:

– When more than 3/4 of the population within a given category chose the response option, that result is communicated by a large green square

– When 1/2 to 3/4 of the population chose an option, this is communicated by a large orange lozenge

– When 1/4 to 1/2 of the population chose an option, this is communicated by a grey disc

– When less than one quarter of the population chose an option, that is communicated by a small red dot

– Absence of reply is represented by a dash - .

Generally, response options are arranged in tables by descending frequency of citation within the ACACON category, followed by descending REGUL frequencies.

### Results and discussion

#### Actual and projected future uses of QSAR

Specialist stakeholders (academics or consultants, regulators, and industry users) were asked whether they now use, or have in the past used/tested the methods, under which conditions, and for which purposes. They were asked to indicate the specific methods, models or software applications used or tested.

The Questionnaire I data discussed below portray effective experience today with applying *in silico* methods, models and software. They also indicate stakeholders’ vision of how these methods will be used tomorrow.

#### QSAR methods: Used or not?

Among respondents to the detailed Questionnaire I, the majority (20) states that they have directly used or tested QSAR methods. Of the 33 respondents in total, a single one (ACACON) claims that this use was “*not successful*”.

All respondents indicate that they have some future plans to test or use QSAR methods.

#### Actual models, methods and software in use

Questionnaire I collected free text responses to the query “*Which models, methods and software have been used or tested?*” Twenty-eight of 33 respondents reply, citing in each case more than one model or software suite.

Table 1 shows the methods cited, arranged by descending frequency of citation among the ACACON respondents, followed by frequency of citation among REGUL, then among INDUS.

**Table 1:** The QSAR models cited by respondents

**Model, method, software that have been used or tested**		**Number of citations**		
		**ACACON**	**REGUL**	**INDUS**
	**Σ**	**(12 of 13 subjects replied)**	**(10 of 12 subjects replied)**	**(6 of 8 subjects replied)**
**OECD QSAR Toolbox**	**16**	7	6	3
**EPI, EPI Suite, EPI Win**	**16**	7	5	4
CAESAR	7	3	2	2
Leadscope	4	3		1
ECOSAR	5	2	3	
SPARC	5	2	3	
Toxtree	5	2		3
Topkat	3	1	2	
DEREK	4	1	1	2
ACD/Tox Suite	3	1	1	1
ToxBoxes	2	1	1	
*EQUATIONS from LITERATURE*	2	1	1	
*OWN INTERNAL MODELS*	2	1	1	
Lazar	2	1		1
MultiCase	1	1		1
ChemAxon Marvin	1	1		
Macromodel	1	1		
Molcode Toolbox	1	1		
OncoLogic	1	1		
Sybyl	1	1		
Danish EPA QSAR database	3		3	
LMC Oasis including CataLogic	3		3	
ChemCan	1		1	
ChemSteer	1		1	
EQC	1		1	
PBKB (SimCYP & MCSim)	1		1	
PBT Profiler	1		1	
SoilFug	1		1	

Overall twenty-eight models, methods or software suites are cited by the population responding to the survey. The three most frequently cited suites are: OECD Toolbox, EPISuite and CAESAR. These three suites are used by all three stakeholder communities. CAESAR is the only software not developed within a regulatory initiative. Each of these three suites is available as freeware. This finding may indicate a preference for free, widely accessible systems.

#### Domains of past application, and the endpoints investigated

Overall, all categories of stakeholder respondents are simultaneously investigating different domains of application. The proportion of actual application by area for the respective stakeholders is shown in Table 2 (ordered by descending importance according to the expressed view by ACACON).

**Table 2:** **Domains of past application of *in silico *** **methods**

**Domains in which *in silico *** **methods are by now applied**	**ACACON**	**REGUL**	**INDUS**
Physico-chemical properties			
Human toxicology			
Environmental fate properties			
Ecotoxicology			

The ACACON community shows the highest rate of application in the area of physico-chemical properties of compounds. REGUL and INDUS appear at this time to be lending somewhat less attention to the assessment of physico-chemical properties through QSAR. INDUS also seems to “lag” behind the other categories in applications to human toxicology and environmental fate properties.

Endpoints that have benefitted from QSAR applications among our population are reported in Table 3. This table does not indicate any hierarchy of endpoints. However, endpoints that were mentioned several times across subjects (within a single stakeholder family) are underlined.

**Table 3:** The QSAR models used by respondents

	**Sample endpoints mentioned from actual applications of QSAR**			
**Stake-holder group**	***Physico-Chemical Properties***	***Human Toxicity***	***Ecotoxicity***	***Environmental Properties***
**ACACON**	Boiling point Vapour pressure	Mutagenicity/ genotoxicity (including Ames, micronucleus, mouse SCE, mouse COMET) Carcinogenicity Teratogenicity Acute toxicity (mammals) Skin irritation, corrosion, or sensitization Eye irritation Endocrine disruption (estrogenbinding, antiadrogenic activity)Reprotox hERG inhibition	Acute aquatic invertebrate toxicity (Daphnia) Acute fish toxicity (fathead minnow, trout) Algae Toxicity Terrestrial Ecotoxicology (bees) Tetrahymena	Bioaccumulation/ BCF Half-life in water/soil Ready biodegradability Abiotic hydrolysis/degradation Koc/soil adsorption
	Water solubility Partition coefficients (LogP/LogD)			
(8 of 13 subjects replied)				
**REGUL**	Partition coefficients (Pow)	Carcinogenicity	Aquatic toxicity	Bioaccumulation/ BCF
(6 of 12 subjects replied)		Reproductive toxicity	Daphnia reproduction	Degradation (DT50)
		Mutagenicity/ genotoxicity (also addressing metabolites)	All ecotox part of EPI Suite (Aquatic toxicity acute andchronic algae fish and daphnia)	
		Teratogenesis		
		Acute toxicity Endocrine disruptions (estrogen and androgen binding)		
		Skin sensitization NOEC		
**INDUS**	Partition coefficients (LogP)	Mutagenicity Genotoxicity Carcinogenicity Teratogenicity	Acute aquatic invertebrate toxicity (Daphnia) (LC50 daphnia magna as supporting information) Fish toxicity	

#### Domains of future application, and the endpoints targeted

All respondents indicated that they have plans for future applications even if they have not yet tested *in silico* methods. Our data suggest overall that all three stakeholder populations today are in a phase of experimentation with QSAR models. Their practice and activity are not “set” at this time.

Regulators overall and within subjects indicated that there would be a slight tendency to transfer or add attention to environmental fate and ecotoxicology endpoints.

#### Functions addressed by past applications

Table 4 considers the functions to which the stakeholders have applied QSAR models.

**Table 4:** **Functions addressed by past applications of *in silico *** **methods**

**Functions addressed by actual (past) applications**	**ACACON**	**REGUL**	**INDUS**
Fast evaluation of the properties of chemicals of interest			
Research and development, for the evaluation of toxicity			
Regulatory requirements - as supporting information			
Regulatory requirements - as part of a weight-of-evidence approach			
Prioritisation of compounds for further analysis			
Regulatory requirements - as the key study			-
None / not sure	-		

ACACON presently appear to prefer QSAR tools as time-saving devices and fundamental research devices. They also acknowledge regulatory data demands.

Among REGUL, QSAR is principally applied to provide supporting information. Use for fast evaluation and as part of the weight-of-evidence approach is also acknowledged.

A non negligible proportion of REGUL states “none/not sure” about the pertinent functions – but an examination of individual data shows these respondents have already tried QSAR methods (generally the OECD Toolbox). This confessed ignorance by regulators regarding functions may simply flag a need to develop their familiarity with the tools.

Among INDUS (like ACACON) fast triage applications are important – more so than in the regulatory community. The profile for prioritisation uses is similar if less pronounced. INDUS and REGUL give comparable importance to the use of QSAR for providing supporting information. INDUS match other stakeholders in their acknowledgement of weight-of-evidence uses, but differ by appearing to leave aside key study applications. INDUS more than REGUL utilize QSAR for research and development for the evaluation of toxicity.

#### Functions addressed in planned (future) applications

The respondents of every category appear to recognize that *in silico* methods will be applied more broadly in the future. Some stakeholders are gearing up to use, test or develop these methods themselves; but even those who today do not plan to use them clearly recognize their growing importance.

Regulators appear to anticipate a slight widening of functionality.

The largest anticipated change will be in more frequent application by regulators for the needs of prioritisation.

#### Perceived benefits and attractions of QSAR methods

Scientists, regulators, and industry users were asked their view on the main reasons to use QSAR methods.

The data in Table 5 give a picture of the differential attractions and benefits of the methods in the perception of these specialist stakeholders.

**Table 5:** Main reasons to use QSAR methods going forward

**Main reasons**	**ACACON**	**REGUL**	**INDUS**
To identify and prioritise substances of concern.			
To improve the response to regulatory requirements such as risk assessment and classification and labelling.			
To reduce the use of vertebrates in experiments - to meet regulatory requirements.			
To reduce the time and costs of experiments.			
To assess potentially thousands of chemicals simultaneously			
To reduce the use of vertebrates in experiments - to meet our own ethical policies.			
To address endpoints for which animal models are not fully accepted.			

There is a primary consensus across stakeholders that going forward, QSAR methods are very attractive for identifying and prioritising substances of concern. For ACACON and REGUL in particular this perception is quite strong.

Also consensual across stakeholders, albeit at a lower level, is the view that these methods offer some attraction for assessing potentially thousands of chemicals simultaneously.

ACACON and REGUL appear quite confident that risk assessment, classification and labelling will be well addressed by QSAR methods, whereas INDUS appear less convinced.

INDUS enthusiastically endorse *in silico* to reduce the laboratory use of vertebrates to meet regulatory requirements, an enthusiasm diminishing somewhat in the view of ACACON and quite modest in the view of REGUL.

One regulator explained:

• *“Reduction of vertebrate testing is a major goal, however, secondary to improved risk management which is the reason why I didn't tick the two boxes [concerned]”.*

The benefit of *in silico* methods to address in-house ethical requirements on animal use is most attractive to industry and ACACON; understandably this feature is less attractive to REGUL.

REGUL find *in silico* methods more interesting than do ACACON and INDUS stakeholders to address endpoints for which animal models are not fully accepted.

Comments from ACACON highlighted two further attractions of *in silico* methods:

• “*To supplement the information level for lower tonnage chemicals with limited test information requirements*”

• “*As a ‘glue’ between in vitro data in ITS approaches*”.

#### Current barriers to the use of QSAR methods

This section reports findings from both Questionnaires I and II.

Specialist stakeholders (consultants, academics, regulators, and industry users) were asked what prevents or limits their use of QSAR methods.

#### Generic barriers

Respondents to the simplified Questionnaire II who have not used QSAR are in fact waiting for a chance to try out the methods. The strong majority said they were “interested” and none was hostile to the methods. Overall the results suggest that persons already interested and whose role allows them to apply the methods may be very open to opportunities to do so.

The varied replies to Questionnaire I as to what prevents or limits the use of *in silico* methods revealed no predominant barrier. Just 3 REGUL, and one ACACON or INDUS, stated that QSAR methods are “*not a priority for his/her organization*”. Only one of 33 respondents expressed preference for traditional, well-known and accepted methods. This striking finding suggests that such a preference is *not* a drag on application of QSAR today. The next section suggests that better information and guidance may be the key to reinforcing *in silico* uses.

#### Needs for information or guidance

Stakeholders indicated areas in which more information or regulatory guidance might overcome barriers to the use of QSAR models. While all categories called for more information or guidance, differential knowledge gaps are described by these specialist stakeholders. Table 6 shows the results about the information needs.

**Table 6:** Needs for information or guidance

**Main option***/sub option*	**ACACON**	**REGUL**	**INDUS**
*All proportions are calculated on total stakeholder subsample*			
**We need more information and/or regulatory guidance** …			
*to assess whether a model can be viewed as scientifically valid and adequately documented;*			
*to be able to use the technical software, and understand the outputs from it;*			
*to know how to integrate different kinds of results from different methods into a submission;*			
*to know what QSAR models are available or appropriate for our work;*			
*to know more about when and how QSAR / in silico methods can be used.*			

Interestingly, REGUL answers focus on pragmatic application. Replies suggest that their primary need is for good grasp of software outputs (the results of the model, and their meaning). To a lesser degree, they need information helping to assess the confidence they may place in QSAR tools, and identification of appropriate uses of the models. ACACON focus more on the scientific aspects. INDUS responses here seem to suggest that they need reassurance on the model acceptability and pointers to the best available models.

#### Opening the way forward to increased use of *in silico* methods

Specialist stakeholders were asked what would help them to use QSAR methods, and what in their view will have most impact on the wider acceptance and use of these methods.

#### What would facilitate use of QSAR*/in silico* methods

In this question stakeholders reported items that could favour more use of QSAR methods. Table 7 shows the results.

**Table 7:** Facilitating the use of QSAR methods

**What would help you to use QSAR / *in silico *** **methods?**	**ACACON**	**REGUL**	**INDUS**
Seeing good examples of industry using *in silico* methods successfully (in documentary video, industry events, online reports and trade magazines).			
Lists and reviews of the available models, with information on where to access them.			
Examples from the regulators about acceptance of / enthusiasm for *in silico* methods			
Seeing more peer-reviewed journal articles about the practical applications of *in silico* methods, illustrated by case studies.			
Clear guidelines for reporting toxicity results from *in silico* methods (maybe as an automatic report generator within the software that matches the submission format).			
Clear standardisation of the ways in which individual QSAR models and their appropriate uses are described, and their applicability domains are defined.			
Support and guidance from laboratories with expertise in the uses of QSARs.			
Examples of the reasoning and transparent documentation required for submissions.			

REGUL clearly place emphasis on aspects that are less outstanding to ACACON or industry. Regulators’ answers point to a desire for a documented, reasoned, exemplified and standardized approach. This is coherent with the results seen above on knowledge needs.

The ACACON sector here places emphasis on pragmatic access to the right tools and demonstration of their successful application.

INDUS’ few replies to these questions seem to align more with ACACON than with REGUL. However, INDUS seem here to be most attracted by a kind of automated, standardized and simplified process. INDUS users made detailed suggestions that highlight the potential value of networking, mutuality of expertise, and cooperation:

• “*Better databases for mining particularly repeat dose endpoints - need agreement on common format - way to deal with proprietary information - resources to shred data and get it entered - broad agreement to collaborate and enter data*”

• “*Definition of expert network in QSAR/in silico methods*”.

#### Views of what will have the most impact on acceptance of QSAR methods

Stakeholders ranked measures that could foster the wider acceptance of QSAR methods (Table 8).

**Table 8:** Measures to foster improved acceptance of QSAR methods

**What will have the most impact on the wider acceptance of QSAR/*in silico *** **methods?**	**ACACON**	**REGUL**	**INDUS**
Industry using *in silico* methods more, and producing high quality results.			
Case study research evidence of the quality and reliability of QSAR methods.			
Use by high-profile companies / organisations, and in cases with high visibility.			
The monitoring, review and updating of models by specialist QSAR laboratories.			
The trademarking of models by trusted software companies or organisations.			-

There is consensus across sectors that in order to foster use of QSAR, industry must demonstrate successful actual applications; ACACON is particularly convinced of this pragmatic assertion. All sectors also affirm that case study evidence of the quality and reliability of *in silico* methods will foster their greater use. In this way, a democratic, evidence-based demonstration, with expert quality assurance in the background, emerges as perhaps the best way to trigger greater use, rather than reliance on high-profile leadership or on trademarking.

### Conclusion and recommendations

The full set of replies to the ORCHESTRA online questionnaires makes it clear that there is no *a priori* refusal of QSAR models. On the contrary, stakeholders in the scientific, regulatory and industry sectors are keen take opportunities to apply *in silico* methods. However, across sectors a considerable need is expressed for “more information or regulatory guidance” on using and applying these methods.

Varying knowledge demands are expressed by the different stakeholders. Scientist and consultant respondents are interested in both technical and scientific aspects of QSAR applications, and they particularly want information that will help to gauge the level of confidence that may be placed in a model. Regulatory respondents on the other hand assign slightly less concern to the scientific validity of models, while requiring relatively more understanding of appropriate applications in view of REACH. Detailed survey results [2] indicate that they seek a good grasp of software outputs (the results of the model, and their meaning). Industry respondents want reassurance that the scientific quality of a given tool is acceptable; next they want pointers to the best available QSAR models. They do not ask for technical information about software, nor for guidance about when and how QSAR can be used.

Given the fact that regulators are the primary actors in acceptance of QSAR models, initiatives should address regulatory information needs as a main target. More detailed attention by developers and consultants to the process requirements of REACH could be valuable. A good model and its suite of tools are not sufficient, if the model is not described and if output components are not transparent. On the basis of these indications, ORCHESTRA, together with the project ANTARES [3], has promoted the web site VEGA (http://www.vega-qsar.eu), which provides a highly detailed explanation on the analysis of model results for a given substance.

At the time of the survey ECHA had published some documents on the use of alternative methods, including QSAR [4]. Questionnaire replies may mean that these documents were not well known, or that they were not yet sufficient. We note that the ECHA guidance documents benefit from updates and that a series of fact sheets is available.

Respondents emphasized the importance of conducting case studies, consolidating experience with model use. Similarly, there is consensus across sectors that industry demonstrating successful actual applications will have a large impact on broadening use of *in silico* methods. “*An important factor in the confidence building is to simply start using the tools, both industry and regulatory bodies, to see for themselves how they work.*” This is indeed a promising direction, and initiatives should address this.

It is important to involve all stakeholders. Networking, mutualising expertise and cooperation are suggested by respondents as aids to bring industry up to speed in applying the methods.

Responding to information demands also means increasing training initiatives. ORCHESTRA is actively working for this; detailed material can be found at the ORCHESTRA web site.

### Endnote

^a^A priori, the questionnaire offered two categories separating academics and consultants. However, on examination of complementary descriptive data provided by persons in these two categories, it became clear that many academics were also consultants in some capacity. It was decided that in context, the more important distinction was indeed the role difference between these intellectual practitioners, those whose primary stake is health protection, and those serving the industrial stake.

## Competing interests

The authors declare no competing interests.

## Authors’ contributions

CM carried out the analyses of the questionnaire survey and produced the report. EB evaluated the regulatory significance of the results. SP co-developed the questionnaire survey. All authors read and approved the final manuscript.

## References

[B1] ORCHESTRA project http://www.orchestra-qsar.eu

[B2] Mays C A report of barriers to disseminating results of environmental research, and stakeholders needs related to the use of in silico models. Report of on-line stakeholder survey 2011 http://www.orchestra-qsar.eu/sites/default/files/ORCHESTRA_Stakeholder-Survey_QSAR-Benefits-Barriers_Report_20110518.pdf

[B3] ANTARES project http://www.antares-life.eu

[B4] ECHA report Guidance on information requirements and chemical safety assessment. Chapter R.6: QSARs and grouping of chemicalshttp://echa.europa.eu/web/guest/guidance-documents/guidance-on-information-requirements-and-chemical-safety-assessment

